# Shotgun-Based Mass Spectrometry Analysis of Phospholipid and Triacylglycerol Molecular Species and Eicosanoids in Salmon Muscle Tissue on Feeding Microbial Oil

**DOI:** 10.3390/md22010011

**Published:** 2023-12-21

**Authors:** JuDong Yeo, Stefanie M. Colombo, Nigel I. Guerra, Christopher C. Parrish

**Affiliations:** 1 Department of Ocean Sciences, Memorial University of Newfoundland, St. John’s, NL A1C 5S7, Canada; niguerra@mun.ca; 2 Department of Food Science and Biotechnology of Animal Resources, Konkuk University, Seoul 05029, Republic of Korea; 3 Department of Animal Science and Aquaculture, Dalhousie University, Truro, NS B2N 5E3, Canada; scolombo@dal.ca

**Keywords:** salmon, microbial oil, phospholipid, triacylglycerol, eicosanoid, PGE2, lipidomics

## Abstract

The continuous growth of aquaculture places a growing demand on alternative sources of fish oil (FO). Certain microorganisms provide a sustainable replacement for FO due to their content of EPA and DHA, which are essential for fish health. Appreciable evidence shows that changes in feeding sources may alter the nutritional components of salmon; however, the influence of diets on lipid species remains unclear. In this study, the identification and semi-quantification of lipid molecular species in salmon muscle during feeding with a microbial oil (MO) were carried out by focusing on triacylglycerol (TAG) and diacyl-phospholipid using shotgun-based mass spectrometry analysis. DHA in the MO diet was efficiently incorporated into phospholipid structures on feeding, followed by accumulation in salmon muscle. The MO diet elevated the level of certain EPA-containing TAGs, such as TAG C52:5 (16:0_16:0_20:5) and TAG C54:6 (16:0_18:1_20:5), indicating that the MO diet may be an excellent source for enhancement of the abundance of ω3 lipids. Further, prostaglandins (PGs) PGE2 and PGF3α were identified and quantified for the first time in salmonid tissue.

## 1. Introduction

Salmon muscle is rich in ω3 fatty acids such as docosahexaenoic acid (DHA) and eicosapentaenoic acid (EPA). Consumption of ω3 fatty acids leads to a variety of health benefits such as reducing cardiovascular disease (CVD) risk, rheumatoid arthritis, and behavioral disorders [1]. Moreover, ω3 fatty acids are heavily involved in a broad spectrum of physiological roles such as neurogenesis, neuroinflammation, and neurotransmission, indicating their essential roles in the development and aging of the brain. An insufficient supply of ω3 fatty acids results in severe mental disorders such as depression, schizophrenia, bipolar disorder, autism, dementia, and attention-deficit/hyperactivity disorder [2].

Shotgun lipidomics is characterized by direct infusion into the mass spectrometer without an HPLC system, and it has been used for the identification and quantification of various lipid species in biological samples. Shotgun lipidomics enables the acquisition of a mass spectrum of samples at a constant concentration of the solution during direct infusion [3]. Thus, it provides unlimited time to operate multiple fragmentation strategies, including precursor ion, product ion, neutral loss (NL) scanning modes, and selected reaction monitoring (SRM) for the identification of target compounds. Moreover, this system provides abundant opportunities to adjust mass analysis conditions such as the ionization condition, gas pressures, and collision energies, which are constrained in the HPLC-MS/MS system due to one-time injection. Due to this advantage, shotgun-based lipidomics has been widely applied in a variety of research areas such as seed lipidomes, cellular lipidomes, plant lipids, lipid peroxidation in redox biology, and others [4,5]. Meanwhile, multiple NL scans enable the confirmation of each segment of structural components of lipid molecules possessing similar substructures such as triacylglycerols (TAGs) and phospholipids (PhLs) [6]. Considerable research has demonstrated the effectiveness of multiple NL and precursor ion scanning for the identification and quantification of PhL and TAG molecules of diverse sample origins, including vegetable oil, ginseng, and turmeric [6,7,8].

Fishmeal and fish oil are traditional ingredients in salmonid feeds, but now they are becoming unsustainable due to their high cost following their limited supply, leading to research and development of appropriate alternatives to overcome this challenge, including heterotrophic protists [9]. Thraustochytrids, which have a uniform spherical shape between 30 and 100 μm in diameter, are classified into a marine group of eukaryotic protists called Labyrinthulomycetes and are abundant in marine ecosystems [10]. Increasing evidence has been reported on the health-promoting potential of microbial DHA and EPA in many clinical trials [11]. Thus, microbial oil from thraustochytrids could be a promising alternative to fish oil due to their high level of ω3 fatty acids. Meanwhile, eicosanoids, derived by enzymatic or non-enzymatic oxidation of arachidonic acid or other PUFAs, are responsible for intercellular signaling, sustaining homeostatic functions, and mediating pathogenic mechanisms in animals [12]. Fish leukocytes synthesize and release prostaglandins (PGs) on immunostimulation and they play a key role in regulating the primary biological functions of fish leukocytes, affecting the innate immune system of fish [13]. Recent research has reported many health-promoting abilities of eicosanoids including suppressing inflammation, neurodegenerative diseases, cancer, central nervous system injury, and neuropsychiatric conditions [14,15,16].

Recently, much research has been carried out on alterations in the nutritional components such as lipids (by focusing on fatty acid profiles), proteins, and micromolecules in salmon muscle tissue as affected by different feeding sources. However, monitoring lipid species such as TAGs, PhLs, and eicosanoids after feeding a different dietary source has rarely been investigated. This is important for consumers because ω3 PUFA bound to phospholipids seems to exert differential bioavailability and biological effects on consumption in comparison to neutral forms of ω3 PUFA [17]. In previous work from our research group, the total lipid content, total TAG, and total PhL in salmon muscle after feeding different diets were measured, and as an extension of that research, this study was designed to observe the detailed composition of TAG and PhL species. In terms of PhLs, alkyl acyl PhLs were not analyzed as they are minor components in salmon muscle tissue [18].

Thus, the objective of the present study was to observe the alteration in the levels of PhL and TAG molecules as well as prostaglandins PGE2 and PGF3α in salmon muscle tissue after feeding four different diets using shotgun-based mass spectrometry (MS) analysis. Here, we focused on tracing the flow of DHA, which is rich in microbial oil, into PhL and TAG in salmon muscle tissue.

## 2. Results

### 2.1. Identification of PhLs in Salmon Muscle Tissue Using MS Analysis

The lipidomics analysis was conducted to determine the major classes of PhLs such as PC, PE, PS, and PI in salmon muscle tissue. The molecular diversity of PhLs with a different combination of fatty acids was determined using ESI-MS analysis. In our previous study, we reported that shotgun lipidomics is a useful means for the identification and quantification of PhL species in salmon muscle tissue, and in the present study, the same approach was applied to investigate the changes in PhL profiles and their contents after feeding different diets [19]. The sodium adducts of PhLs were prepared to elevate the ionization of lipid molecules before injection into the mass spectrometer. Sodium adducts of PhLs in mass spectrometer analysis have been widely used to analyze fatty acids and their oxidation products from preadipocytes, serum, and animal lung tissue [20,21].

**Figure 1:**
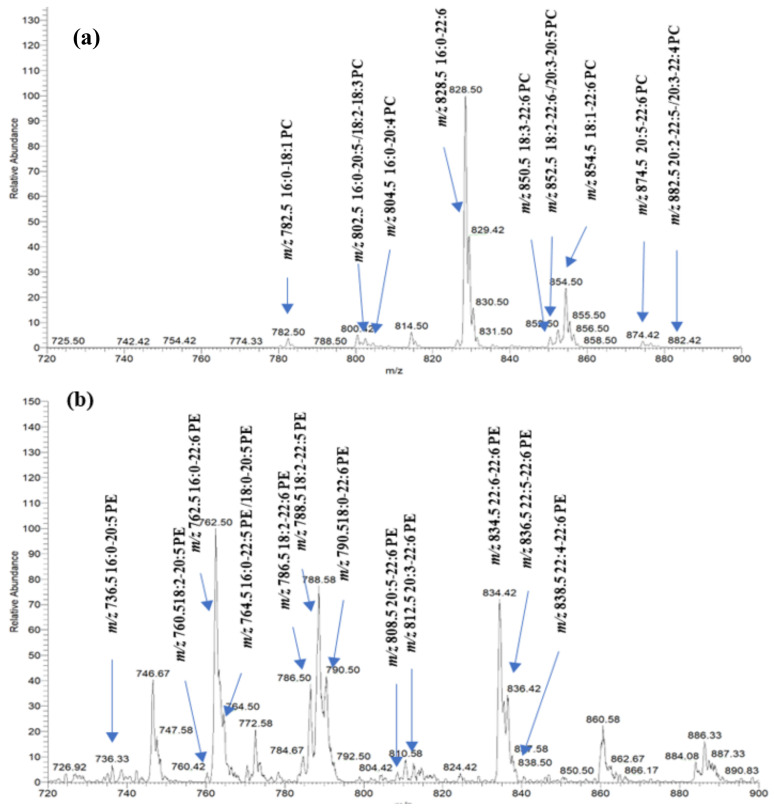
Mass spectra of each class of PhLs in salmon muscle tissue. (**a**) PC species identified in the positive mode at NL 183.1 accounting for the phosphocholine moiety; (**b**) PE species identified in the negative mode at precursor ion scan at 196.0.

Representative mass spectra at the specific NL and precursor ion modes used for the identification of PhL species are presented in Figure 1 along with labels of PCs and PEs identified in salmon muscle tissue.

### 2.2. Changes in the Level of PC, PE, PS, and PI Species in Salmon Muscle Tissue after Being Fed Four Different Diets

**Figure 2:**
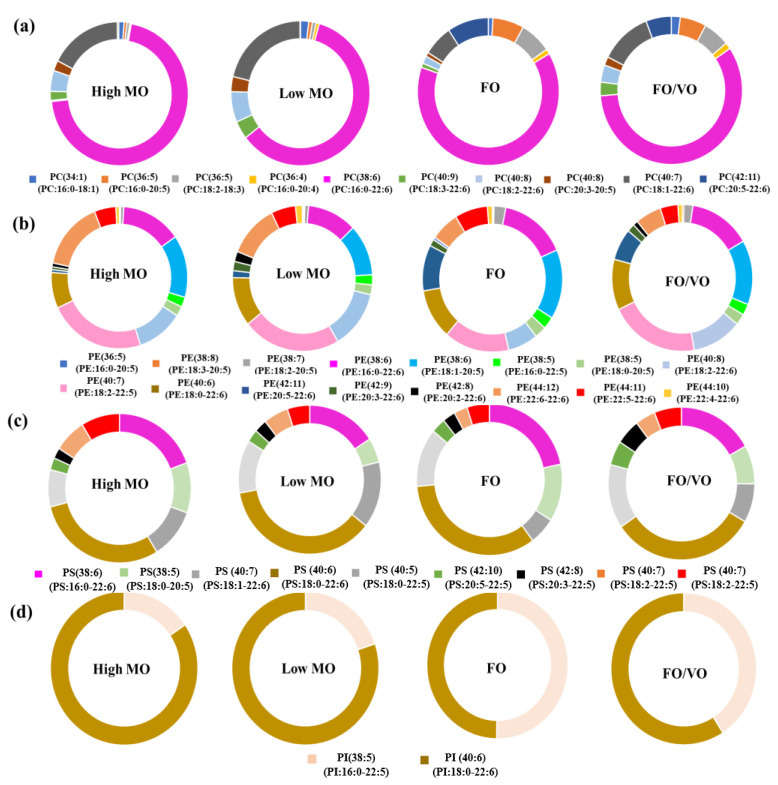
Changes in the level of (**a**) PC, (**b**) PE, (**c**) PS, and (**d**) PI in salmon muscle tissue when fed four different diets (High MO: high microbial oil; Low MO: low microbial oil; FO: fish oil; FO/VO: a mixture of fish and vegetable (canola) oil).

All PC species identified were detected in the form of sodium adducts due to the inclusion of sodium hydroxide before injection into the mass spectrometer. The list of PC species and their changes after feeding four different diets are shown in Figure 2 and Table S1. Shotgun lipidomics using ESI-MS/MS analysis enabled the identification of a total of 10 PC species in the salmon muscle tissue as well as monitoring the alteration of their profiles and levels after feeding four different diets. Most PC species in salmon muscle tissue contained ω3 PUFAs such as DHA and EPA. In particular, DHA was ubiquitous in PC species with 50% of PC species possessing DHA in their chemical structure. The predominant PC species in the salmon muscle tissue was PC38:6 (16:0_22:6-PC) [M + Na]^+^ at *m/z* 828.4, among others. The level of PC38:6 in salmon muscle tissue fed with an FO diet accounted for at least 59.5% of PC species after 16 weeks of feeding. Low MO and FO/VO displayed similar values of 62.2 and 59.5%, respectively. However, the level of PC38:6 fed with high MO, which is more abundant in the level of DHA than other diets, showed a significantly higher value compared to FO and FO/VO diets (*p* < 0.05). This indicates that the high level of DHA in the high MO diet was efficiently incorporated in the form of PhL in the salmon muscle tissue over 16 weeks of feeding. Moreover, principal component analysis (PCA) showed that FO and high MO diets had similar distributions of molecular species, while low MO and FO/VO were different, especially in PC (Figure S1).

**Figure 3:**
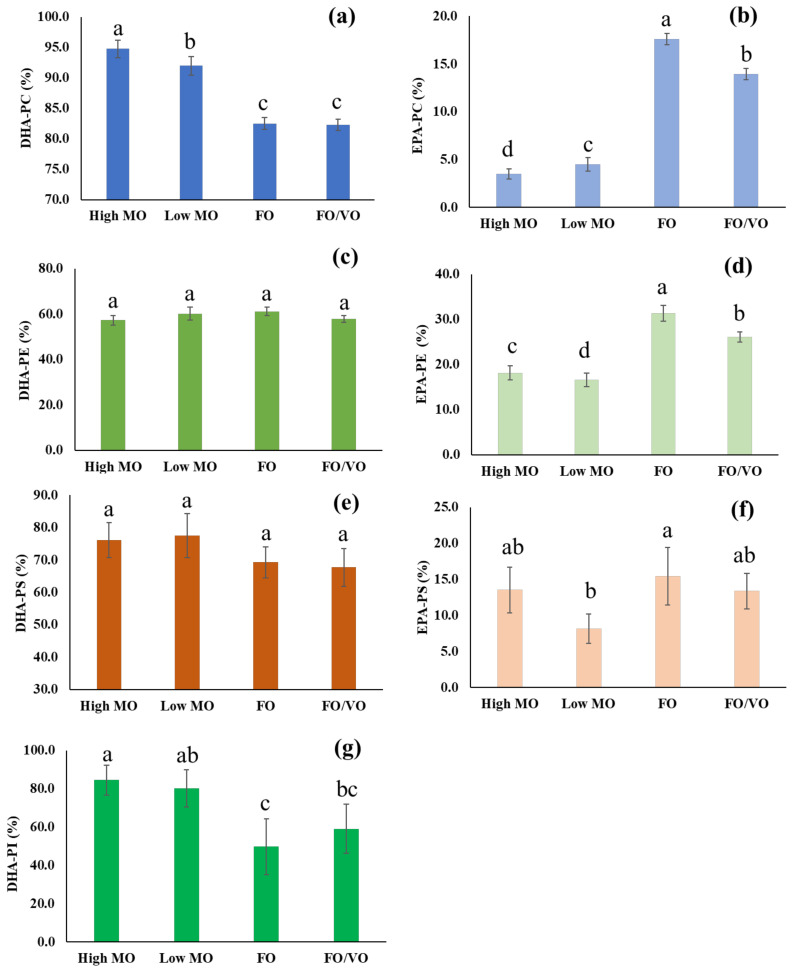
Changes of DHA- and EPA-containing PhLs in salmon muscle tissue on feeding four different diets. (**a**) DHA-containing PC (**b**) EPA-containing PC (**c**) DHA-containing PE (**d**) EPA-containing PE (**e**) DHA-containing PS (**f**) EPA-containing PS (**g**) DHA-containing PI. Data are mean ± s.d., *n* = 6. Values in each panel that have a different letter are significantly different (*p* < 0.05).

The changes in the level of DHA- and EPA-containing PhLs in salmon muscle tissue when fed four different diets are summarized in Figure 3. Feeding high DHA diets such as high MO and low MO significantly increased the level of DHA-containing PCs in the salmon muscle tissue. Feeding a high MO diet enhanced DHA-containing PCs up to 94.8% after 16 weeks of feeding. This value is significantly higher than when an FO diet containing 14.34 mg of DHA/g of oil was fed; it resulted in 82.5% of DHA-containing PCs. In terms of EPA-containing PCs, feeding the FO diet showed a much higher level of EPA-containing PCs (17.6%) compared to high (3.4%) and low MO diets (4.5%), reflecting levels of EPA in each diet (*p* < 0.05). Thus, this result clearly shows that DHA or EPA contents in the diets significantly affect the level of DHA- and EPA-containing PCs in salmon muscle tissue.

The identification and quantification of PE, PS, and PI were carried out in the negative mode by employing multiple precursor ion scans. A total of 16 PE species were found in the salmon muscle tissue using shotgun-based mass spectrometry analysis. Most PE species, except two compounds, contained DHA or EPA in their chemical structure, and this indicates an abundance of those ω3 fatty acids in the salmon muscle tissue in the form of PE. Among others, PE(40:7) (18:2_22:5-PE) [M − H]^−^ at *m/z* 788.5 showed the highest level by accounting for 23.0%. Feeding trials with the different diets did not cause significant changes in the level of DHA-containing PEs as shown in Figure 3; however, it showed a large alteration in the level of PE species such as PE(40:8) (18:2_22:6-PE) [M − H]^−^ at m/z 786.5, PE(42:11) (20:5_22:6-PE) [M − H]^−^ at *m/z* 808.5, and PE(44:12) (22:6_22:6-PE) [M − H]^−^ at m/z 834.5 due to the different diet compositions. In particular, the level of PE(42:11) (20:5_22:6-PE) in the salmon muscle tissue showed a significant difference depending on diets; namely, high MO, low MO, FO, and FO/VO showed 0.6%, 1.5%, 10.2%, and 7.0% in the level of PE(42:11) (20:5_22:6-PE), respectively; thus, FO feeding resulted in 10 fold higher levels compared to high MO feeding (*p* < 0.05). Unlike DHA-containing PEs, the level of EPA in the diets was well reflected in the EPA-containing PEs in the salmon muscle tissue after 16 weeks of feeding; namely, the levels of EPA-containing PEs in the salmon muscle tissue fed with high MO, low MO, FO, and FO/VO were 18.2, 16.6, 31.4, and 26.1%, respectively. Thus, this indicates that the high EPA content in the FO diet was well converted into EPA-containing PEs in the salmon muscle tissue over 16 weeks of feeding.

A total of nine PS and two PI species were identified in the salmon muscle tissue. All nine PS species contained DHA or EPA in their structure. Among others, PS(40:6) (18:0_22:6-PS) [M − H]^−^ at *m/z* 834.5 was the predominant PS species, and its contents after feeding four different diets ranged from 29.7% to 36.5%. However, the level of DHA- and EPA-containing PSs in the salmon muscle tissue did not show a remarkable difference with the different diets. Meanwhile, only two PI species, PI(38:5) (16:0_22:5-PI) [M − H]^−^ at *m/z* 883.5 and PI(40:6) 18:0_22:6-PI [M − H]^−^ at *m/z* 909.5 were found in the salmon muscle tissue.

Meanwhile, the quantitative analysis for the total amount of each subclass of PhL was carried out and summarized in Table S2. Total amounts of PhL in salmon muscle fed high MO, low MO, FO, and FO/VO were 2.49, 2.86, 6.72, and 7.56 mg/g, respectively, showing high values in FO and FO/VO feeding. Among PhL subclasses, PE accounted for a higher proportion compared to other PhL groups (*p* < 0.05).

### 2.3. Changes in the Levels of PUFA-Containing TAG Species in Salmon Muscle Tissue after Being Fed Four Different Diets

**Figure 4:**
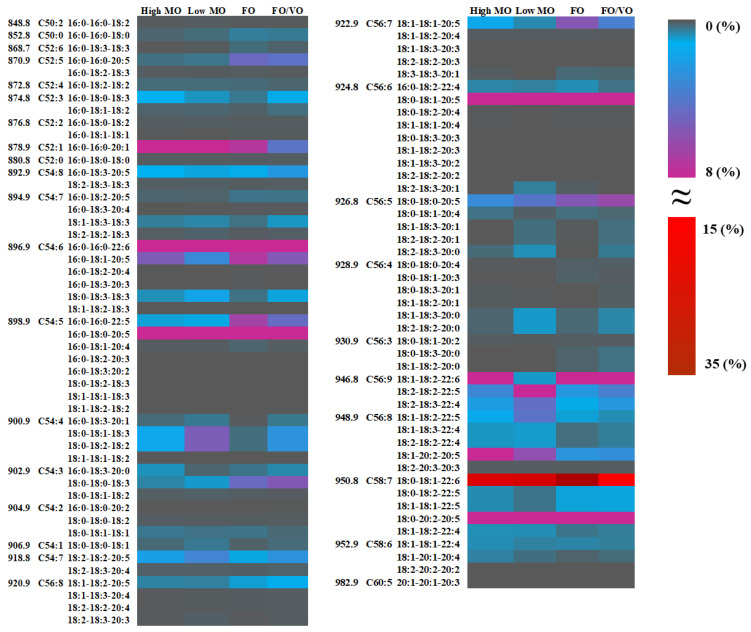
Proportions (% total) of triacylglycerol species in salmon muscle tissue when fed four different diets (High MO: high microbial oil; Low MO: low microbial oil; FO: fish oil; FO/VO: a mixture of fish and vegetable (canola) oil). The numbers in each row indicate *m/z*, C: double bond ratio, and acyl combination of TAG species.

The heatmap of the identified TAG species and their changes after being fed four different diets is shown in Figure 4, and the list of TAG molecules is provided in Table S3. Shotgun lipidomics using ESI-MS/MS analysis enabled the identification of a total of 94 TAG species in salmon muscle tissue. A large proportion of TAG species in salmon muscle tissue possesses ω3 PUFAs such as DHA and EPA. The predominant TAG species in the salmon muscle tissue was TAG(C58:7) (18:0_18:1_22:6) [M + NH_4_]^+^ at *m/z* 950.8, among others, accounting for 25.5–28.3% of the total TAG content. Another predominant TAG molecule was TAG(54:6) (16:0_16:0_22:6) [M + NH_4_]^+^ at *m/z* 896.9. The levels of TAG(54:6) (16:0_16:0_22:6) in salmon muscle fed with high MO, low MO, FO, and FO/VO were 4.20%, 6.14%, 16.59%, and 13.39%, respectively.

**Figure 5:**
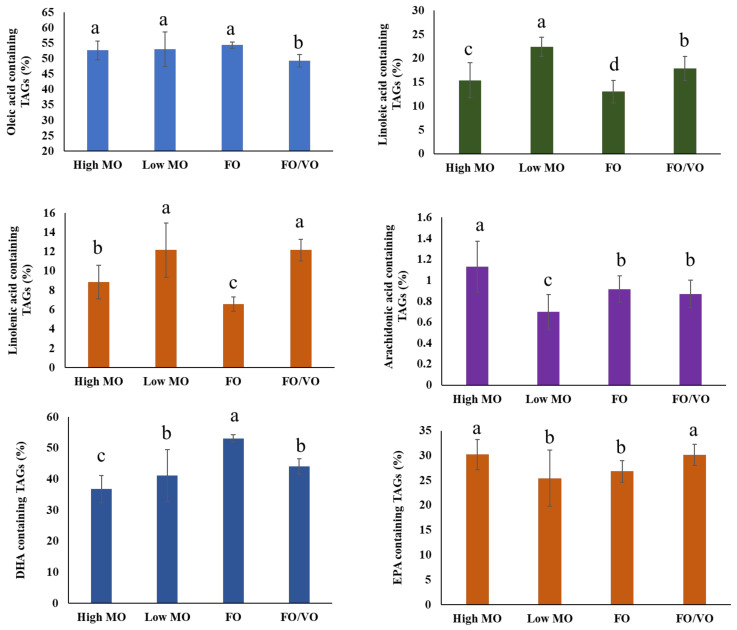
Alterations of oleic acid-, linoleic acid-, linolenic acid-, arachidonic acid-, DHA-, and EPA-containing TAGs in salmon muscle tissue when fed four different diets (High MO: high microbial oil; Low MO: low microbial oil; FO: fish oil; FO/VO: a mixture of fish and vegetable (canola oil). Data are mean ± s.d., *n* = 6. Values in each panel that have a different letter are significantly different (*p* < 0.05).

The alterations in the levels of PUFA-containing TAGs are summarized in Figure 5. The levels of oleic acid-containing TAGs did not show a significant difference among high MO, low MO, and FO treatments, but they did show a lower level with FO/VO feeding. The proportion of linoleic acid in the four different diets (Table S4) is well connected to the level of linoleic acid-containing TAGs in the salmon muscle tissue after being fed over 16 weeks (Figure 5). The levels of linolenic acid in salmon muscle tissue after being fed high MO, low MO, FO, and FO/VO were 3.3%, 4.1%, 1.5%, and 3.8%, respectively, and this also coincides with linolenic acid-containing TAGs in the present study. Overall, the levels of linoleic and linolenic acid in the different diets were well reflected in the linoleic acid- and linoleic acid-containing TAGs in the salmon muscle tissue after being fed for 16 weeks.

Feeding diets containing high DHA levels did not lead to an increase in DHA-containing TAGs in the salmon muscle tissue. For instance, salmon fed high MO showed a lower level of DHA-containing TAGs after being fed for 16 weeks, while FO diets, with the second lowest amount of DHA, resulted in a higher level of DHA-containing TAGs. This implies that feeding a high level of DHA-containing diets did not directly lead to the increase in DHA-containing TAGs in the salmon muscle tissue. A similar trend was also found in EPA-containing TAGs. The EPA content in the four diets did not significantly influence the accumulation of EPA-containing TAGs in the salmon muscle tissue, except for low MO, which had the lowest level of EPA-containing TAGs. However, EPA-containing TAG molecules showed a different trend even with the same diet feeding.

### 2.4. Identification of Eicosanoids and Their Quantitative Changes in the Salmon Muscle Tissue When Fed Four Different Diets

Eicosanoids, which are derived from the enzymatic or non-enzymatic oxidation of arachidonic acid or other PUFAs, are responsible for intercellular signaling, sustaining homeostatic functions, and mediating pathogenic mechanisms in animals [12]. Thus, eicosanoids play a significant role in the metabolism of fish and other animal systems.

In our previous work, the optimization of MS analysis for the identification of PGE2 and PGF3α in salmon muscle tissue was carried out using product ion scanning in the negative mode. The product ions of standards were compared with those from salmon muscle tissue for the identification of PGE2 and PGF3α by adopting the optimized ESI-MS/MS analysis condition as reported in the previous work.

The changes in the level of PGE2 in salmon muscle tissue when fed four different diets are described in Table 1. The level of PGE2 in salmon muscle tissue fed with FO and FO/VO showed significantly higher values at 47.1 and 35.8 ng/g compared to high MO (14.6 ng/g) and low MO (7.1 ng/g) (*p* < 0.05). The alteration in the level of PGF3α in salmon muscle tissue was also measured after being fed the four different diets. Feeding with FO showed a significantly higher level of PGF3α in salmon muscle tissue compared to that of other treatments such as high MO, low MO, and FO/VO (*p* < 0.05) (Table 1).

## 3. Discussion

In the present study, the changes in PhL species in salmon muscle tissue were investigated using MS analysis. In particular, the alteration of DHA-containing PhLs using the MO diets was the focus. Overall, DHA in the MO diets was efficiently incorporated into the PhLs, especially in PC species, in the salmon muscle tissue as shown in the results.

Broughton et al. [22] reported that feeding genetically modified (GM) oilseeds to Atlantic salmon significantly changed lipid profiles in their brain, eyes, gills, intestines, and liver. Tissue-dependent trends were found in the incorporation of long chain-polyunsaturated fatty acid (LC-PUFA) as well as the putative alteration in stereospecific isomer numbering (*sn*). This indicates that changes in oil sources for fish feeding could lead to notable alterations in lipid composition in fish tissues, which agrees with the present study. Jin et al. [23] investigated how diet affects lipid profiles in salmon muscle tissues and found comparable changes in PhL profiles driven by the switch in diet, but more dramatic alteration was observed in the gut and liver.

Meanwhile, Wang and Zhang [23] investigated the phospholipid profiles of muscle from *Ctennopharyngodon idellus* and they found that it contains 16:0_18:1-PC, 16:0_20:4-PC, and 18:0_20:5-PC as the predominant PC species, which is different to salmon muscle with 16:0-22:6-PC as the primary PC species. Moreover, Inhamuns and Bueno Franco [24] reported that phospholipids in Mapara muscle tissue possess high levels of 16:0 and 18:1. Thus, the above findings reveal significant differences in fatty acid and phospholipid compositions of muscle tissue of different fishes.

Lipids in dietary sources may change their structural formation in the digestion and absorption process, that is, TAG molecules in the dietary source are digested into *sn*-2-monoacylglycerol and free fatty acids, followed by the absorption and re-synthesize into PhL or other lipids [25]. Thus, the remodeling of lipidomes in salmon muscle tissue on feeding different diets may occur in this process. The alteration of lipidomes in salmon muscle can also be caused by the influence of feeding diets on membrane-associated protein. Jeromson et al. [26] reported that EPA influences the alterations of membrane-associated proteomes, which are related to the synthesis and modification of lipids, in muscle cell models, followed by changes in membrane lipid species. Thus, the EPA content in four different diets may change the composition of lipid molecules in salmon muscle tissue by affecting membrane-associated proteomes.

The levels of DHA and EPA may play crucial roles in maintaining a well-balanced metabolism and suppressing severe diseases in the human body. Thus, the incorporation of DHA in salmon muscle tissue via MO diets would be an excellent strategy for enhancing the nutritional and functional value of Atlantic salmon for consumers. For instance, DHA has been proven to be an effective bioactive compound in enhancing heart and brain function as well as retarding the rate of cognitive decline [27]. Meanwhile, a substantial body of evidence has reported the beneficial health effects of EPA such as reducing cardiovascular disease (CVD), asthma, rheumatoid arthritis, behavioral disorders, cystic fibrosis, etc. [28]. Some animal studies demonstrated that marine ω3 fatty acids elevated the level of the anti-inflammatory cytokine IL-10 [29]. Several studies show that marine ω3 fatty acid supplements reduced the production of TNF, IL-6, and IL-1β by endotoxin-stimulated monocytes in healthy humans [30]. Robinson and Stone [31] summarized the remarkable antiatherosclerotic and antithrombotic activities of ω3 fatty acids in animal and human studies.

The forms in which DHA and EPA are consumed are of particular interest. The elevated level of DHA in salmon muscle tissue, as found in the PhL profile, on feeding a high DHA diet such as high MO may provide a variety of beneficial effects on human health for consumers. Calder [32] reported the anti-inflammatory effect of ω3 fatty acids in the form of phospholipids in marine species such as salmon. Hals et al. [33] demonstrated that purified phospholipids rich in ω3 fatty acids significantly reduced cardiovascular disease risk factors. Shiels et al. [34] reported that phospholipids containing ω3 fatty acids exhibit strong anti-inflammatory and antithrombotic effects. PhLs containing ω3 fatty acids derived from salmon displayed high antithrombotic activities against the platelet-activating factor (PAF) and thrombin pathways [18]. Further, recent studies demonstrated that DHA-PhLs in particular show a higher absorption and bioavailability in the brain compared to that of DHA-TAG, leading to the prevention of Alzheimer’s disease [35]. Thus, it is important to achieve such enrichment in the PhLs of the fish by feeding them appropriate diets such as MO.

Meanwhile, DHA and EPA levels in the diets did not significantly affect the levels of DHA- and EPA-containing TAGs in salmon muscle tissue. Hixson et al. [36] demonstrated that salmon fed with camelina oil, which contains a low DHA content (0.5–1.1% of total fatty acids), sustained DHA levels in salmon muscle tissue and led to the increase in ω3 and ω3 intermediary biosynthesis products. This fact indicates the endogenous production of 22:6ω3 from dietary 18:3ω3 in salmon muscle, and it supports the results of the present study. PCA analysis displayed a similar distribution of TAG molecules with each dietary treatment, although high MO samples appeared to be more concentrated on the positive side of the first axis, while low MO samples appeared more concentrated in the upper left quadrant (PCA results not shown). Jin et al. [25] investigated the alteration of TAG molecules in salmon muscle tissue when fed different diets in which TAG molecules with higher chain lengths (more than 60) and double bonds between 12 and 18 were highly related to being fed fish oil. On the other hand, being fed vegetable oil (VO) was closely associated with the TAG species possessing shorter carbon chain lengths (less than 60) and low unsaturation (i.e., 56:10, 54:10, and 58:10). However, they did not report the alteration in lipid species (i.e., 18:0_18:2_22:6, 18:1_18:2_22:4, 16:0_18:2_20:5, etc.), limiting the comparison with the present study in monitoring TAG species during feeding trials. Until now, most research has not been focused on the analysis of TAG molecules; thus, the present study may fill the gaps in understanding lipid species in salmon muscles and their changes.

The discrepancy in PGE2 contents between FO and MO diets might be due to the difference in the levels of their precursor molecule, 20:4ω6, required for their synthesis. As shown in Table S4, the levels of 20:4ω6 in high MO, low MO, FO, and FO/VO were 0.44, 0.43, 1.85, and 1.41 mg/g, respectively. Thus, the higher levels of PGE2 in the salmon muscle tissue fed with FO and FO/VO compared to MO diets might be due to higher contents in their precursor molecules (20:4ω6) in the diets.

PGE2 plays a significant role in modulating signaling, maintaining homeostasis, and facilitating pathogenic mechanisms in animals; there are health-beneficial effects in reducing neurodegenerative diseases, cancer, central nervous system injury, and neuropsychiatric conditions as well [12]. Thus, PGE2 in salmon muscle tissue is a significant molecule in sustaining the metabolism and homeostasis of humans and animals.

In terms of PGF3α, given that the FO diet contains high EPA, the precursor molecule for the biosynthesis of PGF3α, as shown in Table S4, the high level of PGF3α in salmon muscle tissue after FO feeding might be due to a direct dietary effect. However, this pattern was not well reflected in the high MO diet, which gave the second highest PGF3α concentration in salmon muscle tissue even with the low level of dietary EPA. Therefore, as mentioned in the PGE2 section, this result indicates that there are other indirect factors influencing the biosynthesis of PGF3α in salmon muscle tissue.

The techniques used in the present study would be a useful means for analyzing lipid species in a variety of foods, in particular, monitoring the changes in the PhL and TAG molecules as affected by food processing. As proven through the present study, shotgun-based mass spectrometry analysis displayed an effective performance in the identification and quantification of TAG species containing specific fatty acyl chains (i.e., DHA, EPA, ARA, etc.); thus, this high throughput can be widely utilized in exploring other lipid species such as sterol ester, wax ester, sphingomyelin (SM), cardiolipin (CL), and galactosylceramide (GalCer) in diverse plant- and animal-based foods.

Although shotgun-based lipidomics is a high throughput approach with numerous advantages, classical shotgun lipidomics has some limitations including ambiguous identification of isobaric lipid molecules, the presence of nominal mass, matrix effect, isomers that generate almost identical fragmentation patterns, and fatty acids with other linkages [37,38]. Thus, we are planning to carry out additional research to address the above drawbacks of shotgun-based lipidomics as well as TLC and chemical quantifications for PC, PE, PI, and PS and following comparisons with HPLC-MS/MS.

## 4. Materials and Methods

### 4.1. Materials

The microbial oil (MO) isolated from *Schizochytrium* sp. thraustochytrids was provided by Mara Renewables Corporation (Dartmouth, NS, Canada). Standards, including 18:1-18:1-phosphatidylcholine, 18:1-18:1-phosphatidylethanolamine, 16:0-16:0-phosphatidylserine, and 18:1-18:1-phosphatidylinositol, used for the identification and quantification of phospholipid species were obtained from Sigma-Aldrich Canada Ltd. (Oakville, ON, Canada). PGE2 and PGF3α standards were purchased from Cayman Chemical Company (Ann Arbor, MI, USA). Sodium hydroxide, chloroform (HPLC grade), and methanol (HPLC grade) were obtained from Fisher Scientific Co. (Nepean, Ottawa, ON, Canada).

### 4.2. Experimental Fish

Preparation of experimental diets including high MO, low MO, fish oil (FO), and the mixture of fish and canola oil (FO/VO), experimental design, growth performance, and sampling procedures were carried out as summarized by Wei et al. [9] Briefly, salmon (~25 g initial weight) were fed experimental diets in triplicate tanks for 16 weeks in freshwater (12 °C, 100% oxygen saturation) and were hand fed to satiation twice daily. Their initial lengths were from 13.3 to 16.2 cm, and their final lengths ranged from 21.3 to 22.4 cm, showing approximately 8.0 cm of growth over 16 weeks. Two fish per tank, a total of six fish per group, were euthanized by injecting an overdose of buffered tricaine methane sulfonate (TMS) at a concentration of 400 mg/L, and their clinical death was ensured before sampling. The ethical treatment of salmon used in this research followed guidelines as described by the Canadian Council of Animal Care. The skin was removed on the left side and white dorsal muscle tissue was extracted and immediately frozen and stored at −80 °C. The fatty acid content of the experimental diets is provided in Table S4.

### 4.3. Lipid Extraction

The procedure for the extraction of crude lipids in salmon muscle tissue was conducted as described by Parrish [39], with minor modifications. Briefly, 250 mg of salmon muscle tissue, 2 mL of ice-cold chloroform, and 1 mL of methanol were mixed in a 15 mL glass vial and then homogenized using a blender. The metal rod of the blender was subsequently washed with 0.5 mL of chloroform-extracted water and 1 mL of chloroform: methanol (2:1). The mixture was then sonicated for 4 min in an ice bath and centrifuged at 3500× *g* for 2 min. A double pipetting technique was used to transfer the organic layer (bottom) containing lipophilic molecules to a clean glass vial using glass Pasteur pipettes. This procedure was repeated three times, and all organic layers were combined into the vial, followed by removing organic solvent using nitrogen evaporation. Finally, the remaining crude lipid in the vial was re-dissolved with chloroform and kept at −20 °C until further use.

### 4.4. ESI-MS/MS Analysis for the Identification and Quantification of PhLs

The identification of PhLs and their semi-quantitative analysis in salmon muscle tissue was conducted using a triple quadrupole mass spectrometer (TSQ Quantis™ Triple Quadrupole Mass Spectrometer, Thermo Fisher Scientific, Waltham, MA, USA) after electrospray ionization (ESI) as described by Yeo and Parrish [19]. Briefly, the lipid extract of salmon muscle tissue was diluted with 1:4 chloroform: methanol (*v*/*v*) to prepare the final solution at a concentration of 25 mg/mL, followed by the inclusion of 10 μL of sodium hydroxide in the sample solution for the formation of sodium adducts of PhLs. The sample solution was injected directly into the mass spectrometer using a high-pressure syringe pump (F100T2, Chemyx Inc., Stafford, TX, USA) at a flow of 10 μL/min. The analysis of phosphatidylcholine (PC) species was carried out in the positive mode, and the conditions of the mass spectrometer were set to 325 °C ion transfer tube temperature, 4000 (v) ion spray voltage, 10 (arbitrary) aux gas, 50 (arbitrary) sheath gas, 1 (arbitrary) sweep gas, and 30 °C vaporizer temperature. The identification of the PC moiety and two fatty acids of PCs was carried out in NL scanning mode, and for the collision energy, collision-induced dissociation (CID) gas was set at 37 eV and 1 mTorr. The identification of phosphatidylethanolamine (PE), phosphatidylserine (PS), and phosphatidylinositol (PI) was carried out in the negative mode along with multiple precursor ion scans. The conditions for mass spectrometry analysis were set as follows: 325 °C ion transfer tube temperature, 2500 (v) ion spray voltage, 13.8 (arbitrary) aux gas, 2.0 (arbitrary) sheath gas, 0.8 (arbitrary) sweep gas, and 30 °C vaporizer temperature. The same condition of collision energy and CID gas was used for the MS/MS analysis.

Multiple NL scans were utilized in the positive mode for the identification of PC species. The NL at 183.1 accounts for the loss of phosphatidylcholine moiety in the chemical structure of PCs, and other NL corresponding to different fatty acids (i.e., NL 256.4 for palmitic acid, NL 328.4 for DHA, NL 302.4 for EPA, etc.) were employed for the identification of target PC species in the salmon muscle tissue. On the other hand, the identification of PEs, PSs, and PIs was carried out through the precursor ion scan mode in the negative mode.

For the semi-quantitative analysis, isotopic deconvolution was carried out to obtain the corrected peak area of lipids as described by Li [4], that is, it was carried out by applying the following formula Z_M_ = [(NC × 1.12) × (NC × 1.12)/200 + (No × 0.204)]/100. Here, Z_M_ indicates the isotopic correction factor, which is further used to yield the corrected peak signal below. N_C_ and N_O_ denote the numbers of carbon and oxygen atoms in the product ion of the diacylglycerol generated in the fragmentation of molecule M, implying that the number of carbons and oxygens in the lipid species affects the intensities of isotopic signals. Then, the corrected peak signal (^C^I_M+2_), in which M + 2 indicates a molecule having two more molecular mass than molecule M and (^C^I_M+2_) means the corrected intensity of molecule M + 2, was deduced through the formula ^C^I_M+2_ = I_M + 2_ − ^C^I_M_ × Z_M_, in which I_M+2_ indicates the raw signal intensity (before isotopic deconvolution) of lipid species, and ^C^I_M_ denotes the corrected signal of lipid molecule M. The corrected peak signals of the PhL species were utilized for the calculation of their final quantification using external standards including 18:1_18:1-PC, 18:1_18:1-PE, 16:0_16:0-PS, and 18:1_18:1-PI. The equation deduced by a standard curve of 18:1_18:1-PC in an NL scan at 183.1 in the positive ion mode was used for the calculation of the level of PC species in salmon muscle tissue. The same approach was used for the calculation of the level of PE, PS, and PI by applying precursor ion scanning at 196.0, 153.0, and 241.0 in the negative ion mode, respectively.

### 4.5. ESI-MS/MS Analysis for the Identification and Quantification of TAG Species

The identification and semi-quantitative analysis of TAGs using a triple quadrupole mass spectrometer were carried out as described by Yeo and Parrish [19]. Briefly, 16 mM ammonium acetate was added to the lipid extract (final concentration) to form an ammonium adduct, and then 500 µL of the lipid extracts were introduced directly into the mass spectrometer at a flow rate of 10 μL/min. The identification of TAG species and their quantitative analysis were conducted in the positive mode with the following mass spectrometer conditions: 5000 (v) ion spray voltage, 0.4 (arbitrary) sweep gas, 16.3 (arbitrary) aux gas, 30 °C vaporizer temperature, and 325 °C ion transfer tube temperature. The collision energy for the fragmentation of molecular ions was set at 28 eV.

The identification of TAG molecules was carried out by confirming three fatty acid moieties of TAGs using the multiple NL scans, followed by data processing using Xcalibur Software (Version 4.3, Thermo Fisher Scientific, Waltham, MA, USA).

A standard curve of triolein (18:1_18:1_18:1) was created for the quantification of the TAG species identified. The correction of each peak area was carried out by calculating isotopic deconvolution as described by Li [4] as explained in the PhL semi-quantification section. Then, those corrected peak areas were applied to the equations of the standard curve of triolein, followed by the quantification of TAG species. In NL scanning mode, fatty acids have different instrumental responses, in particular the higher signal intensities for DHA and EPA. Thus, in the present study, triolein was chosen for the quantitative analysis due to its average NL response among other TAG molecules. The identification of TAG species was carried out as described in the phospholipid section using multiple NL scans.

### 4.6. ESI-MS/MS Analysis for the Identification and Quantification of PGE2 and PGF3α

Prior to the analysis of PGE2 and PGF3α for creating a standard curve and running samples, the optimization process for searching for the best analysis parameters of the mass spectrometer was carried out. Then, the optimized conditions were applied to the analysis of standards and samples under the same environment. The identification of PGE2 and PGF3α was carried out in the negative mode along with the following analysis conditions: 4490 (v) ion spray voltage, 4.0 (arbitrary) sheath gas, 6.1 (arbitrary) aux gas, 7.1 (arbitrary) sweep gas, 18.26 eV of collision energy, ion transfer tube temperature (325 °C), and vaporizer temperature (30 °C). The quantitative analysis of PGE2 and PGF3α was conducted by using external standards.

### 4.7. Statistical Analysis

Independent replicates (duplicate) for three tanks, representing six salmon per group, were used to carry out statistical analyses, and those values were used for normality tests and one-way ANOVA. Each mean was for a total of six samples per treatment and was analyzed using Tukey’s HSD test (*p* < 0.05) in SPSS 16.0 (SPSS Inc., Chicago, IL, USA). PCA analysis was also carried out using the SPSS package.

## 5. Conclusions

In the present study, we investigated the changes in PhLs, TAGs, PGE2, and PGF3α in Atlantic salmon muscle tissue when fed four different diets using shotgun-based ESI-MS/MS analysis. Here, we found that the DHA present in the diets was efficiently incorporated into PhLs and certain TAGs, followed by accumulation in salmon muscle tissue. In addition, the alteration of PGE2 in salmon muscle tissue was measured after the same dietary treatments. PGE2 content in the salmon muscle tissue displayed a close relationship with the level of precursor molecules (arachidonic acid, 20:4ω6) in each diet. Given that DHA plays a crucial role in the metabolism and physiological actions of fish, the use of microbial oil would be an excellent alternative for fish oil in aquaculture. To the best of our knowledge, this is the first research that applied mass spectrometry-based lipidomics to monitor the alteration of DHA- and EPA-containing TAG molecules in salmonoid tissue. This study provides useful information on the alterations of lipidomes in salmon muscle tissue affected by different dietary sources. The present study focused on the identification and quantification of triacylglycerols and phospholipids (diacyl-phospholipids) as they are major lipid groups among others in salmon muscle tissue accounting for approximately 95% or more. Thus, minor lipids such as alkyl acyl phospholipids were excluded from the target list. However, in future studies, we plan to conduct untargeted lipidomics, which comprehensively observes all lipids, rather than targeted lipidomics as shown in this study.

## Figures and Tables

**Table 1:** Changes in the level of PGE2 and PGF3α in salmon muscle tissue when fed four different diets (High MO: high microbial oil; Low MO: low microbial oil; FO: fish oil; FO/VO: a mixture of fish and vegetable (canola) oil) for 16 weeks. Data are mean ± s.d., *n* = 6.

	High MO	Low MO	FO	FO/VO
PGE2 (ng/g)	14.6 ± 3.1 ^b^	7.1 ± 2.7 ^b^	47.1 ± 8.7 ^a^	35.8 ± 10.6 ^a^
PGF3α (µg/g)	28.2 ± 6.6 ^b^	2.0 ± 0.7 ^c^	36.6 ± 4.9 ^a^	21.1 ± 4.0 ^b^

Values in each row that have the same letter are not significantly different (*p* > 0.05).

## Data Availability

Data are contained in the paper and the Supplementary Information.

## Supplementary Materials

The following supporting information can be downloaded at: https://www.mdpi.com/article/10.3390/md22010011/s1, Table S1: Changes in the level of phospholipid species in salmon muscle tissue upon being fed four different diets; Figure S1: Loading plots of PCA analysis from mass spectrometry analysis of PC and PE; Table S2: Quantitative analysis of total PhL contents in salmon tissue upon being fed different diets (mg/g); Table S3: Alterations in the level of triacylglycerol species in salmon muscle tissue upon being fed four different diets; Table S4: Fatty acid composition (mg/g) of four different diets.
