# Supplementary material for: Shotgun-Based Mass Spectrometry Analysis of Phospholipid and Triacylglycerol Molecular Species and Eicosanoids in Salmon Muscle Tissue on Feeding Microbial Oil
Source: Mar Drugs. 2023 Dec 21;22(1):11. doi: 10.3390/md22010011 (PMC10820676; doi:10.3390/md22010011)
Supplement: Supplementary file 1 [file marinedrugs-22-00011-s001.zip › marinedrugs-2574240-supplementary.pdf]

Table S1. Changes in the level of phospholipid species in salmon muscle tissue upon feeding four different diets

$[M + Na]^+$	C: double bond ratio	PC	High MO (%)	Low MO (%)	FO (%)	FO/VO (%)
782.4	34:1	16:0_18:1-PC	1.3 ± 0.3b	2.0 ± 0.8a	1.1 ± 0.3b	2.1 ± 0.5a
802.4	36:5	16:0_20:5-PC/ 18:2_18:3-PC	1.4 ± 0.4c	1.8 ± 0.8c	14.4 ± 0.8a	12.2 ± 2.0b
804.5	36:4	16:0_20:4-PC	0.1 ± 0.3c	0.7 ± 0.9b	1.1 ± 0.1ab	1.4 ± 0.4a
828.4	38:6	16:0_22:6-PC	72.3 ± 3.7a	62.2 ± 4.6bc	64.4 ± 1.8b	59.5 ± 2.6c
850.4	40:9	18:3_22:6-PC	2.1 ± 0.6c	4.2 ± 0.7a	0.9 ± 0.3d	3.0 ± 0.2b
852.4	40:8	18:2_22:6-PC/ 20:3_20:5-PC	4.8 ± 1.0b	7.0 ± 1.0a	1.8 ± 0.4d	3.8 ± 0.4c
854.4	40:7	18:1_22:6-PC	17.6 ± 1.7b	21.9 ± 1.2a	6.8 ± 1.8d	12.0 ± 1.1c
874.4	42:11	20:5_22:6-PC	0.3 ± 0.3c	0.1 ± 0.4c	9.5 ± 0.8a	5.9 ± 0.4b

$[M - H]^-$		PE	High MO (%)	Low MO (%)	FO (%)	FO/VO (%)
736.5	36:5	16:0_20:5-PE	0.1 ± 0.1b	0.3 ± 0.1a	0.1 ± 0.0b	0.0 ± 0.0c
758.5	38:8	18:3_20:5-PE	0.2 ± 0.1b	0.3 ± 0.1ab	0.3 ± 0.2ab	0.4 ± 0.2a
760.5	38:7	18:2_20:5-PE	0.8 ± 0.4b	0.9 ± 0.6b	2.7 ± 1.1a	2.1 ± 1.0a
762.5	38:6	16:0_22:6-PE/ 18:1_20:5-PE	28.4 ± 7.0ab	22.8 ± 5.8b	30.8 ± 4.8a	28.4 ± 2.4ab
764.5	38:5	16:0_22:5-PE/ 18:0_20:5-PE	4.4 ± 1.4a	4.4 ± 1.8a	5.4 ±1.4a	5.0 ± 1.6a
786.5	40:8	18:2_22:6-PE	11.0 ± 3.5a	13.0 ± 4.6a	6.9 ± 2.1b	11.5 ± 2.7a
788.5	40:7	18:2_22:5-PE	23.0 ± 4.4a	22.6 ± 7.4a	15.0 ± 3.2b	20.6 ± 5.0ab
790.5	40:6	18:0_22:6-PE	8.2 ± 2.3a	10.9 ± 4.3a	11.1 ± 2.2a	11.2 ± 2.0a
808.5	42:11	20:5_22:6-PE	0.6 ± 0.2c	1.5 ± 0.7c	10.2 ± 2.9a	7.0 ± 1.6b
812.5	42:9	20:3_22:6-PE	0.6 ± 0.3b	2.0 ± 1.2a	1.5 ± 0.6a	1.8 ± 0.4a
814.5	42:8	20:2_22:6-PE	0.9 ± 0.3b	2.3 ± 1.0a	0.6 ± 0.2b	1.1 ± 0.5b
834.5	44:12	22:6_22:6-PE	15.7 ± 2.7a	11.7 ± 5.0b	6.6 ± 1.8c	6.0 ± 1.5c

836.5	44:11	22:5_22:6-PE	5.1 ± 1.5b	5.7 ± 2.9ab	7.6 ± 1.6a	4.1 ± 1.4b
838.5	44:10	22:4_22:6-PE	0.8 ± 0.6b	1.6 ± 0.8a	1.2 ± 0.4ab	1.0 ± 0.3ab
**$[M - H]^-$**		**PS**	**High MO (%)**	**Low MO (%)**	**FO (%)**	**FO/VO (%)**
806.5	38:6	16:0_22:6-PS	19.5 ± 7.8a	15.8 ± 7.3a	21.8 ± 9.1a	16.9 ± 10.0a
808.5	38:5	18:0_20:5-PS	10.9 ± 4.8a	5.4 ± 2.5b	12.2 ± 6.5a	8.2 ± 2.5ab
832.5	40:7	18:1_22:6-PS	11.0 ± 6.9ab	14.5 ± 8.2a	5.9 ± 3.2b	8.2 ± 4.0ab
834.5	40:6	18:0_22:6-PS	29.7 ± 6.5a	36.5 ± 11.5a	33.7 ± 8.1a	32.1 ± 9.2a
836.5	40:5	18:0_22:5-PS	8.1 ± 3.0a	11.5 ± 5.0a	12.5 ± 5.5a	13.6 ± 8.4a
854.5	42:10	20:5_22:5-PS	2.6 ± 1.5b	2.8 ± 1.5b	3.2 ± 1.5b	5.2 ± 2.5a
858.5	42:8	20:3_22:5-PS	2.3 ± 1.2b	2.8 ± 2.3ab	2.8 ± 2.4ab	5.4 ± 4.9a
878.5	44:12	22:6_22:6-PS	7.3 ± 1.9a	5.7 ± 3.9ab	3.0 ± 1.4c	4.5 ± 2.2bc
880.5	44:11	22:5_22:6-PS	8.7 ± 3.9a	5.1 ± 2.9b	5.0 ± 2.1b	6.0 ± 4.1ab
**$[M - H]^-$**		**PI**	**High MO (%)**	**Low MO (%)**	**FO (%)**	**FO/VO (%)**
883.5	38:5	16:0_22:5-PI	15.5 ± 7.8b	19.9 ± 9.8b	50.2 ± 14.6a	41.0 ± 12.8a
909.5	40:6	18:0_22:6-PI	84.5 ± 7.8a	80.1 ± 9.8a	49.8 ± 14.6b	59.0 ± 12.8b

Values in each row having the same letter are not significantly different ($p > 0.05$).

Figure S1:. Loading plots of PCA analysis from mass spectrometry analysis of PC and PE (PS and PI were not included due to their limited number).

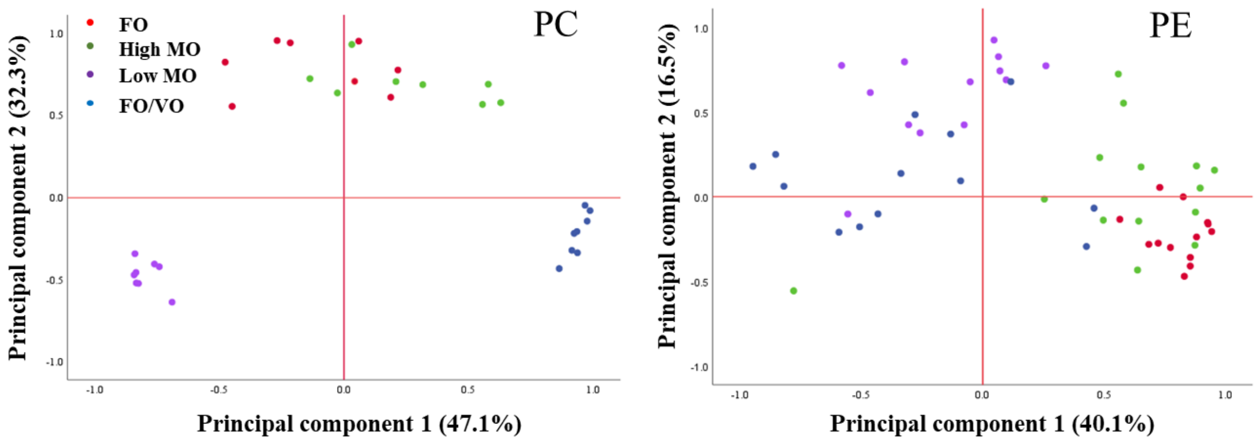

Table S2: Quantitative analysis of total PL contents in salmon tissue upon feeding different diets (mg/g)

	**High MO**	**Low MO**	**FO**	**FO/VO**
PL subclass				
**PC**	0.62 ± 0.10c	0.60 ± 0.05c	1.01 ± 0.09a	0.80 ± 0.05b
**PE**	1.40 ± 0.39c	1.77 ± 0.45c	5.06 ± 1.31b	6.37 ± 0.64a
**PS**	0.37 ± 0.09b	0.41 ± 0.12ab	0.52 ± 0.13a	0.33 ± 0.06b
**PI**	0.10 ± 0.05ab	0.08 ± 0.02b	0.13 ± 0.04a	0.06 ± 0.02b
**Total**	2.49	2.86	6.72	7.56

Values in each row having the same letter are not significantly different (p > 0.05).

Table S3: Alterations in the level of triacylglycerol species in salmon muscle tissue upon feeding four different diets

m/z $[M + NH_4]^+$	C: double bond ratio	Acyl combination	High MO (%)	Low MO (%)	FO (%)	FO/VO (%)
848.8	C50:2	16:0_16:0_18:2	0.03 ± 0.01a	0.05 ± 0.03a	0.03 ± 0.01a	0.03 ± 0.01a
852.8	C50:0	16:0_16:0_18:0	0.14 ± 0.14b	0.23 ± 0.18ab	0.44 ± 0.29a	0.39 ± 0.16a
868.7	C52:6	16:0_18:3_18:3	0.02 ± 0.02c	0.04 ± 0.03bc	0.24 ± 0.11a	0.11 ± 0.09b
870.9	C52:5	16:0_16:0_20:5/ 16:0_18:2_18:3	0.32 ± 0.11c	0.38 ± 0.05c	2.12 ± 0.15a	1.97 ± 0.23b
872.8	C52:4	16:0_18:2_18:2	0.22 ± 0.05a	0.22 ± 0.10a	0.21 ± 0.09ab	0.15 ± 0.07b
874.8	C52:3	16:0_18:0_18:3/ 16:0_18:1_18:2	1.15 ± 0.31a	0.85 ± 0.23b	0.47 ± 0.15c	1.20 ± 0.33a
876.8	C52:2	16:0_18:0_18:2/ 16:0_18:1_18:1	0.07 ± 0.04a	0.08 ± 0.04a	0.08 ± 0.02a	0.05 ± 0.01a
878.9	C52:1	16:0_16:0_20:1	15.02 ± 3.17a	7.99 ± 6.15b	2.81 ± 1.46c	1.92 ± 0.86c
880.8	C52:0	16:0_18:0_18:0	0.05 ± 0.07a	0.05 ± 0.05a	0.06 ± 0.07a	0.06 ± 0.03a
892.9	C54:8	16:0_18:3_20:5/ 18:2_18:3_18:3	1.08 ± 0.23b	0.96 ± 0.37b	1.01 ± 0.29b	1.42 ± 0.16a
894.9	C54:7	16:0_18:2_20:5/ 16:0_18:3_20:4/ 18:1_18:3_18:3/ 18:2_18:2_18:3	0.66 ± 0.20b	0.77 ± 0.26b	0.68 ± 0.12b	1.13 ± 0.23a
896.9	C54:6	16:0_16:0_22:6/ 16:0_18:1_20:5/ 16:0_18:2_20:4/ 16:0_18:3_20:3/ 18:0_18:3_18:3/ 18:1_18:2_18:3	7.10 ± 1.56c	8.92 ± 3.29c	19.38 ±0.98a	16.58 ± 1.89b
898.9	C54:5	16:0_16:0_22:5/ 16:0_18:0_20:5/ 16:0_18:1_20:4/	5.39 ± 0.65d	7.14 ± 1.08b	6.95 ± 0.48c	8.85 ± 0.73a
900.9	C54:4	16:0_18:3_20:1/ 18:0_18:1_18:3/ 18:0_18:2_18:2/	2.45 ± 0.34c	4.85 ± 0.57a	0.52 ± 0.07d	3.24 ± 0.55b
902.9	C54:3	16:0_18:3_20:0/ 18:0_18:0_18:3/ 18:0_18:1_18:2	1.23 ± 0.22b	1.02 ± 0.34b	2.43 ± 0.74a	2.91 ± 0.69a
904.9	C54:2	16:0_18:0_20:2/ 18:0_18:0_18:2/ 18:0_18:1_18:1	0.42 ± 0.27a	0.34 ± 0.33a	0.37 ± 0.17a	0.24 ± 0.15a
906.9	C54:1	18:0_18:0_18:1	0.19 ± 0.14ab	0.38 ± 0.43a	0.10 ± 0.04b	0.22 ± 0.12ab

918.8	C54:7	18:2_18:2_20:5/ 18:2_18:3_20:4	1.36 ± 0.39a	1.75 ± 0.63a	1.00 ± 0.16b	1.57 ± 0.31a
920.9	C56:8	18:1_18:2_20:5/ 18:1_18:3_20:4/ 18:2_18:2_20:4/ 18:2_18:3_20:3	0.52 ± 0.23c	0.61 ± 0.18c	0.93 ± 0.18b	1.10 ± 0.16a
922.9	C56:7	18:1_18:1_20:5/ 18:1_18:2_20:4/ 18:3_18:3_20:1	1.23 ± 0.25c	0.61 ± 0.25d	2.58 ± 0.24a	1.93 ± 0.20b
924.8	C56:6	16:0_18:2_22:4/ 18:0_18:1_20:5/ 18:0_18:2_20:4/ 18:1_18:1_20:4/ 18:2_18:3_20:1	6.12 ± 0.76a	4.48 ± 1.03b	6.40 ± 0.64a	5.14 ± 0.26b
926.8	C56:5	18:0_18:0_20:5/ 18:0_18:1_20:4/ 18:1_18:3_20:1/ 18:2_18:2_20:1/ 18:2_18:3_20:0	2.04 ± 0.41d	3.08 ± 0.52b	2.96 ± 0.50c	3.55 ± 0.23a
928.9	C56:4	18:0_18:0_20:4/ 18:0_18:1_20:3/ 18:0_18:3_20:1/ 18:1_18:2_20:1/ 18:1_18:3_20:0/ 18:2_18:2_20:0	1.11 ± 0.15c	1.57 ± 0.40b	4.04 ± 0.11a	1.31 ± 0.16b
930.9	C56:3	18:0_18:1_20:2/ 18:0_18:3_20:0/ 18:1_18:2_20:0	0.05 ± 0.00c	0.04 ± 0.00c	0.31 ± 0.13b	0.58 ± 0.33a
946.8	C56:9	18:1_18:2_22:6/ 18:2_18:2_22:5/ 18:2_18:3_22:4	7.14 ± 2.18b	11.18 ± 1.46a	6.63 ± 1.29b	8.34 ± 1.98b
948.9	C56:8	18:1_18:2_22:5/ 18:1_18:3_22:4/ 18:2_18:2_22:4/ 18:1_20:2_20:5/ 18:2_20:3_20:3	6.03 ± 0.50a	5.93 ± 0.62a	2.80 ± 0.18c	3.03 ± 0.59b
950.8	C58:7	18:0_18:1_22:6/ 18:0_18:2_22:5/ 18:1_18:1_22:5/ 18:0_20:2_20:5/ 18:1_18:2_22:4	38.73 ± 4.63a	36.00 ± 5.91a	37.31 ± 1.41a	32.18 ± 1.51b
952.9	C58:6	18:1_18:1_22:4/ 18:1_20:1_20:4	1.08 ± 0.19a	0.74 ± 0.17b	0.64 ± 0.18b	0.66 ± 0.18b

Values in each row having the same letter are not significantly different (p > 0.05).

Table S4: Fatty acid composition (mg/g) of four different diets (This information is obtained from Wei et al. 2021)

Fatty acid	High MO	Low MO	FO	FO/VO
14:0	8.61 ± 1.44a	5.57 ± 1.52b	10.87 ± 0.89a	7.51 ± 0.51ab
15:0	1.19 ± 0.24a	0.75 ± 0.20b	0.84 ± 0.10ab	0.59 ± 0.02b
16:0	29.44 ± 6.01b	24.40 ± 6.44c	32.15 ± 2.96a	28.57 ± 1.79b
17:0	0.34 ± 0.08c	0.24 ± 0.07c	0.81 ± 0.08a	0.58 ± 0.06b
18:0	3.97 ± 0.85b	4.86 ± 1.26b	7.18 ± 0.62a	7.43 ± 0.34a
16:1n-7	5.48 ± 1.00c	4.30 ± 1.18c	12.21 ± 1.10a	9.18 ± 0.54b
16:1n-9	0.13 ± 0.05b	0.18 ± 0.04b	0.49 ± 0.07a	0.49 ± 0.08a
18:1n-9	57.74 ± 13.29ab	87.60 ± 23.50a	22.93 ± 1.97b	74.86 ± 4.47a
16:3n-4	0.13 ± 0.08c	0.08 ± 0.07c	2.08 ± 0.22a	1.37 ± 0.09b
18:2n-6	27.82 ± 6.79ab	37.54 ± 9.91a	14.40 ± 1.35b	33.62 ± 2.04a
18:3n-6	0.15 ± 0.05b	0.10 ± 0.06b	0.42 ± 0.09a	0.27 ± 0.09ab
20:4n-6	0.44 ± 0.13b	0.43 ± 0.12b	1.85 ± 0.31a	1.41 ± 0.12a
18:3n-3	6.99 ± 1.75a	10.53 ± 2.80a	2.13 ± 0.43b	8.79 ± 0.55a
18:4n-3	0.30 ± 0.06c	0.25 ± 0.09c	3.53 ± 0.42a	2.44 ± 0.15b
20:4n-3	0.36 ± 0.08bc	0.19 ± 0.10c	1.02 ± 0.21a	0.76 ± 0.26ab
20:5n-3	1.59 ± 0.30c	1.44 ± 0.54c	22.96 ± 2.26a	15.44 ± 1.00b
21:5n-3	0.00 ± 0.01c	0.01 ± 0.01c	0.97 ± 0.05a	0.66 ± 0.02b
22:5n-6	5.29 ± 1.26a	3.04 ± 0.78b	0.47 ± 0.11c	0.38 ± 0.11c
22:6n-3	29.41 ± 7.35a	17.28 ± 4.78b	14.34 ± 1.44b	9.83 ± 0.45b

Values in each row having the same letter are not significantly different ($p > 0.05$).
